# First step to facilitate long-term and multi-centre studies of shear wave elastography in solid breast lesions using a computer-assisted algorithm

**DOI:** 10.1007/s11548-017-1596-3

**Published:** 2017-05-06

**Authors:** Katrin Skerl, Sandy Cochran, Andrew Evans

**Affiliations:** 1 Medical Research Institute, Ninewells Hospital and Medical School, Mailbox 4, Dundee, DD1 9SY Scotland, UK; 2 Image Science for Interventional Techniques, University of Auvergne, 28, Place Henri Dunant, BP 38, 63001 Clermont-Ferrand Cedex, France; 3 School of Engineering, University of Glasgow, Glasgow, G12 8QQ Scotland, UK

**Keywords:** Computer-aided diagnosis, Breast cancer, Shear wave elastography, Ultrasound, Data assessment, Diagnosis

## Abstract

**Purpose:**

Shear wave elastography (SWE) visualises the elasticity of tissue. As malignant tissue is generally stiffer than benign tissue, SWE is helpful to diagnose solid breast lesions. Until now, quantitative measurements of elasticity parameters have been possible only, while the images were still saved on the ultrasound imaging device. This work aims to overcome this issue and introduces an algorithm allowing fast offline evaluation of SWE images.

**Methods:**

The algorithm was applied to a commercial phantom comprising three lesions of various elasticities and 207 in vivo solid breast lesions. All images were saved in DICOM, JPG and QDE (quantitative data export; for research only) format and evaluated according to our clinical routine using a computer-aided diagnosis algorithm. The results were compared to the manual evaluation (experienced radiologist and trained engineer) regarding their numerical discrepancies and their diagnostic performance using ROC and ICC analysis.

**Results:**

ICCs of the elasticity parameters in all formats were nearly perfect (0.861–0.990). AUC for all formats was nearly identical for ${E}_{\mathrm{max}}$ and ${E}_{\mathrm{mean}}$ (0.863–0.888). The diagnostic performance of SD using DICOM or JPG estimations was lower than the manual or QDE estimation (AUC 0.673 vs. 0.844).

**Conclusions:**

The algorithm introduced in this study is suitable for the estimation of the elasticity parameters offline from the ultrasound system to include images taken at different times and sites. This facilitates the performance of long-term and multi-centre studies.

## Introduction

**Fig. 1:**
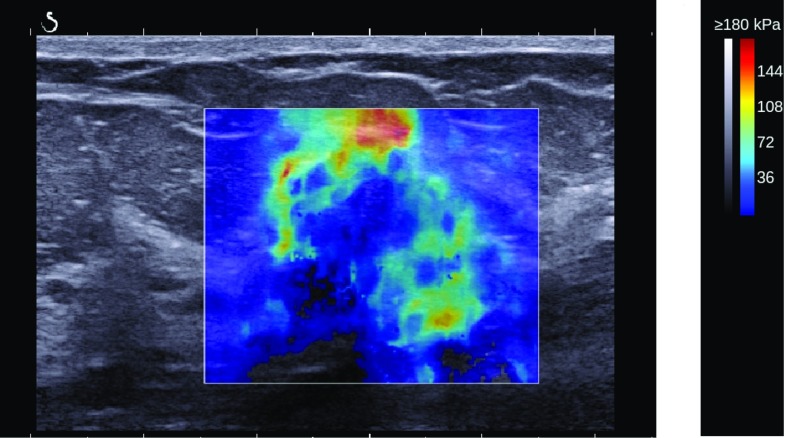
SWE image of a malignant solid breast lesion. The elasticity values are represented as a colour map overlaying the greyscale B-mode ultrasound image

Shear wave elastography (SWE) is an ultrasound imaging modality which visualises the elasticity of tissue. It was introduced by Bercoff et al. [1] and has been in clinical use since 2009 [2]. During observations, the propagation speed of the shear wave is measured and the elasticity, represented by Young’s Modulus, E, is calculated by the ultrasound device. The elasticity is visualised as a colour map overlaying the greyscale B-mode ultrasound image of the lesion (Fig. 1).

Several studies have shown that adding the evaluation of the elasticity of a lesion with SWE to B-mode ultrasound assessment according to the Breast Imaging Reporting and Data System (BI-RADS) [3] is helpful for the differentiation of benign from malignant lesions [2, 4–6] as malignant tissue is generally stiffer than benign tissue [7]. Berg et al. [2] recommend to use a cut-off threshold for the maximum elasticity of ${E}_{\mathrm{max}}=80~\hbox {kPa}$ for improving benign/malignant differentiation of BI-RADS 3 and 4a lesions, whereas Evans et al. [8] recommend a threshold, ${E}_{\mathrm{mean}}=50~\hbox {kPa}$, relating to the mean elasticity. To the best of our knowledge, all previously introduced methodologies evaluating the quantitative SWE parameters maximum elasticity (${E}_{\mathrm{max}}$), mean elasticity (${E}_{\mathrm{mean}}$) and standard deviation (SD) are only applicable to images still stored at the ultrasound device. Older images are automatically deleted from the device, in our clinic after about 3 months.

Presently, to determine the elasticity parameters, in our clinic a circular region of interest (ROI) is placed manually over the stiffest part of the tissue where ${E}_{\mathrm{mean}}$ over all pixels included in the ROI is maximal as examined by the operator. The ROI is positioned initially at a stiff region as suggested by the colour coding and then shifted manually while monitoring ${E}_{\mathrm{mean}}$. This procedure is time-consuming and can be done only directly on the ultrasound system and not on remote work stations. Hence, computer- aided estimation of elasticity parameters (a form of computer-aided diagnosis—CAD) would be helpful.

CAD of breast lesions is possible for mammography, ultrasound and magnet resonance imaging (MRI) and is routine in many mammography practices [9–13]. Moon et al. [14, 15] introduced an algorithm for CAD of strain elastography which evaluates the ratio of stiff and compliant tissue pixels and hence does not provide quantitative image analysis in the same way as SWE. Xiao et al. [16, 17] recently introduced CAD for SWE. However, they evaluated the performance of their algorithm by comparing it to the BI-RADS classification of the greyscale ultrasound images and not to pathology. As BI-RADS tends to declassify high-grade cancers [18] while SWE tends to declassify low-grade cancers [19], it is difficult to evaluate the performance of the algorithm introduced by Xiao. Nevertheless, several other approaches introduced CAD of SWE as [20, 21]. Lo et al. [20] evaluate the histograms of the RGB (red green blue) images and correlate them with malignancy, whereas Zhang et al. [21] evaluate the texture of the elastograms. Acharya et al. [22] apply three levels of discrete wavelet transform, while Zhang et al. [23] build a deep learning architecture in their later work to introduce novel methodologies to assess the lesions’ malignant potential. Skerl et al. [24] evaluate the qualitative pattern distribution and not the quantitative SWE parameters. Thus, neither CAD algorithm applies the clinical routine, evaluating the SWE parameters ${E}_{\mathrm{max}}$, ${E}_{\mathrm{mean}}$ and SD, and direct clinical application is difficult.

The aim of the present work is to provide and validate an easy and reproducible algorithm which enables the evaluation of the elasticity parameters on remote work stations from images obtained using the standard settings (50% opacity, blue to red “jet” colour coding; 0–180 kPa, red representing stiff tissue) which are most commonly used in clinical practice. To enable remote image evaluation, the SWE images need to be first saved from the device. The user has the option to choose between the DICOM and JPG saving format. Thus, within this work we also investigated the influence by the saving format onto the accuracy of the automatic image assessment. The algorithm also applies the clinical routine for quantitative SWE and positions the ROI to ascertain the elasticity values automatically. Overall, this algorithm enables evaluation of SWE images obtained at various times and sites. Hence, this algorithm enables long-term studies and facilitates multi-centre studies assuring a standardised SWE evaluation. Furthermore, the algorithm reduces bias by human evaluators who can be influenced by the greyscale appearance of the lesion.

## Materials and methods

### Breast elasticity phantom

**Fig. 2:**
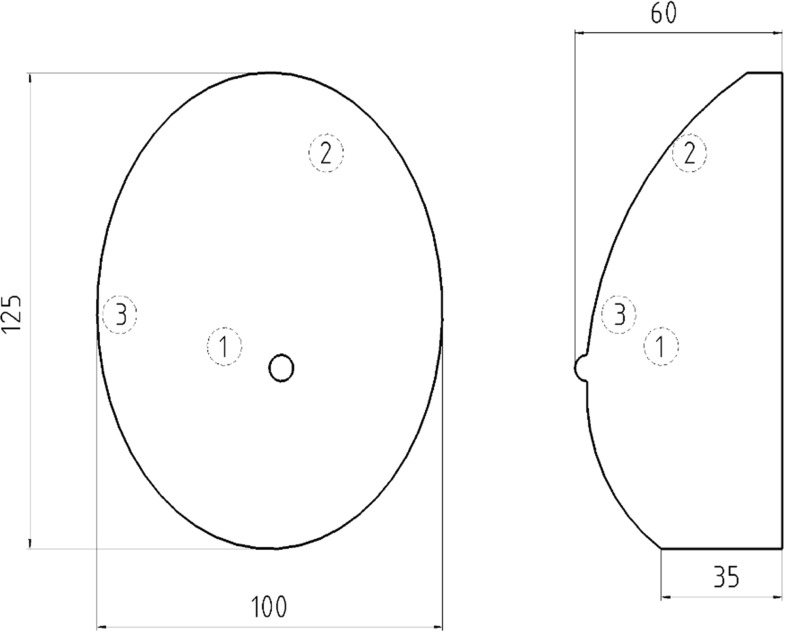
Diagram of the phantom with the three evaluated inclusions. Measurements in mm

A commercial breast elasticity phantom from CIRS (Model 059, Norfolk, VI, USA) was used. The phantom is made of the specific hydrogel material Zerdine® [25] and comprised three inclusions evaluated in this work (Fig. 2). Each inclusion was spherical, with a diameter of 11 mm. The three inclusions were measured by the inexperienced observer, as detailed below. In the following text, they are denoted as Inclusion 1, Inclusion 2 and Inclusion 3.

### In vivo study-group

The study-group comprised 207 consecutive solid breast lesions (70 benign, 137 malignant) in 203 patients (age range 21–92 years, mean 57.9 years) who underwent core biopsy or surgical excision and were imaged in our clinic between September 2012 and April 2013. There were no exclusion criteria. The study-group contained screen-detected lesions and symptomatic patients. Ethical approval by the National Research Ethics Service guidance was not necessary [26]. Written informed consent for the use of images in our research was obtained, as is standard procedure in our clinic.

### Ultrasound system

All images were acquired with the Aixplorer ultrasound imaging system (Product version 6.3.0, SuperSonic Imagine, Aix-en-Provence, France). The ultrasound probe has a frequency range 4–15 MHz with axial resolution 0.3–0.5 mm and lateral resolution 0.3–0.6 mm. The same probe was used to obtain the B-mode and SWE images.

SWE images were obtained using the standard settings: elasticity range 0–180 kPa, red being the stiffest values, opacity of 50%. Each lesion was imaged in two orthogonal planes with two images obtained in each plane. The four values were averaged for each lesion.

### Manual image evaluation

For all measurements, a ROI with a diameter of 2 mm was used. The ROI was positioned by the individual observers at the stiffest point in the image in terms of ${E}_{\mathrm{mean}}$. The elasticity parameters ${E}_{\mathrm{max}}$, ${E}_{\mathrm{mean}}$ and SD were evaluated from the ROI. The measurements were done directly with the system by two independent observers, one radiologist with experience of more than 20 years in breast ultrasound and more than 2 years experience in the performance of SWE, and the other observer, the engineer developing the algorithm trained to exclude artefacts and the pectoral muscle but with no experience in breast ultrasound.

### Automatic image evaluation

The images were saved on the imaging system in three different formats: DICOM (Digital Imaging and Communications in Medicine), JPG (Joint Photographic Experts Group) and QDE (quantitative data export; a format distributed by SuperSonic Imagine which allows the direct export of the elasticity values for research only and not available on the commercial products). We did not change the pre-settings including the compression rate to increase applicability to the clinical procedure. In clinical routine, the pre-settings are rarely changed, and thus, the pre-settings can be assumed to be the most common settings applied.

The automated algorithm was implemented using the same routine as for the manual evaluation to enable clinical compatibility and using the image processing software MATLAB ([27], MathWorks, Natwick, MA, USA). The elasticity image is surrounded by a white frame. Thus through detection of this frame, the elasticity image can be segmented.

**Fig. 3:**
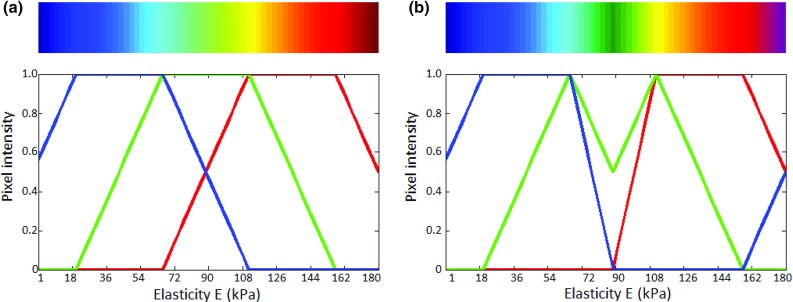
Colour maps shown with corresponding numerical values; **a** standard “jet” colour map from MATLAB; **b** SWE colour map using an opacity of 50%

**Fig. 4:**
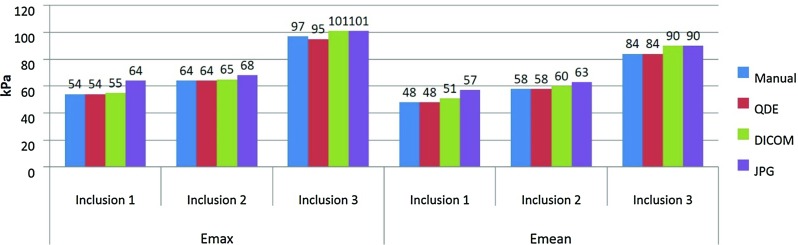
Averaged estimated values for ${E}_{\mathrm{max}}$ and ${E}_{\mathrm{mean}}$ in the phantom. The elasticity was higher for DICOM and JPG images than for QDE images or manual estimation

The elasticity of the tissue is visualised as a colour map, as shown in Fig. 1. Red represents maximum stiffness, whereas blue represents soft tissue. Hence, the colour map is similar to the “jet” colour map (Fig. 3a) used in image processing software tools such as MATLAB [27]. In the standard system settings, the opacity of the SWE image is set to 50% so that the greyscale B-mode image is still visible underneath. Hence, the “jet” colour map is not directly applicable and an adjusted colour map is needed. Comparing the directly exported elasticity values from the QDE images with the DICOM and JPG images, an adjusted colour map was derived, as shown in Fig. 3b. Using this novel colour map, all DICOM and JPG images ever stored from the device such as images stored on the picture archiving and communications system (PACS) are convertible into SWE values. The elasticity values were calculated from the DICOM and JPG images and elasticity maps were obtained. To apply the standard routine, first of all a circular mask with a ROI of 2 mm is used. This ROI is shifted across the SWE image sequentially with Emean calculated for each ROI and compared to the other results to find the stiffest point of ${E}_{\mathrm{mean}}$. For this ROI, the elasticity parameters ${E}_{\mathrm{mean}}$, ${E}_{\mathrm{max}}$ and SD are calculated. The stiffest ROI is also represented graphically on the elasticity map for manual evaluation to enable the exclusion of artefacts. If the ROI is correctly positioned (as determined by the inexperienced observer), all values were saved to a spread sheet. A further description of the algorithm can be found in [28].

As the colour map ranges from 0 to only 180 kPa, while the elasticity range of the system ranges from 0 to 300 kPa (manual observation and evaluation through QDE format), values higher than 180 kPa were set to 180 kPa (${E}_{\mathrm{max}}$: 53 of the 207 lesions, ${E}_{\mathrm{mean}}$: 29 of the 207 lesions).

### Statistics

The diagnostic performance of the different CAD estimations was compared with web-based software using Chi-square test (SISA, Quantitative Skills, Hilversum, Netherlands). The null hypothesis was rejected at a level of 5% ($p \le 0.05$).

Receiver operator curve (ROC) analysis and intraclass correlation (ICC) evaluation were performed using IBM SPSS (version 22, IBM, Armonk, New York, USA).

## Results

### Results in breast mimicking phantom

The averaged estimated values for ${E}_{\mathrm{max}}$ and ${E}_{\mathrm{mean}}$ shown in Fig. 4a, b indicate that values estimated from the QDE images have very good agreement with the manual estimation (inexperienced observer). However, values estimated from DICOM images are, on average, about 2 kPa (${E}_{\mathrm{max}}$) and 4 kPa (${E}_{\mathrm{mean}}$) stiffer than the manual estimation and values from JPG images are, on average, 6 kPa (${E}_{\mathrm{max}}$) and 7 kPa (${E}_{\mathrm{mean}}$) stiffer than the manual estimation. SD was not analysed for the lesions due to the artificial homogeneity of the phantom.

**Table 1:** Diagnostic performance for all formats

Analysis	Se			Sp			DA		
	${E}_{\mathrm{max}}$	${E}_{\mathrm{mean}}$	SD	${E}_{\mathrm{max}}$	${E}_{\mathrm{mean}}$	SD	${E}_{\mathrm{max}}$	${E}_{\mathrm{mean}}$	SD
Inexperienced	85	91	77	70	54	**77**	82	80	77
Experienced	85	**93**	**84**	76	59	67	80	80	78
CAD: DICOM	90	**96**	**69**	66	54	**60**	82	82	66
CAD: QDE	88	95	74	71	54	77	82	81	75
CAD: JPG	91	**99**	77	63	37	76	81	78	76

Statistic significant values are highlighted in bold

*Se* sensitivity, *Sp* specificity, *DA* diagnostic accuracy

**Fig. 5:**
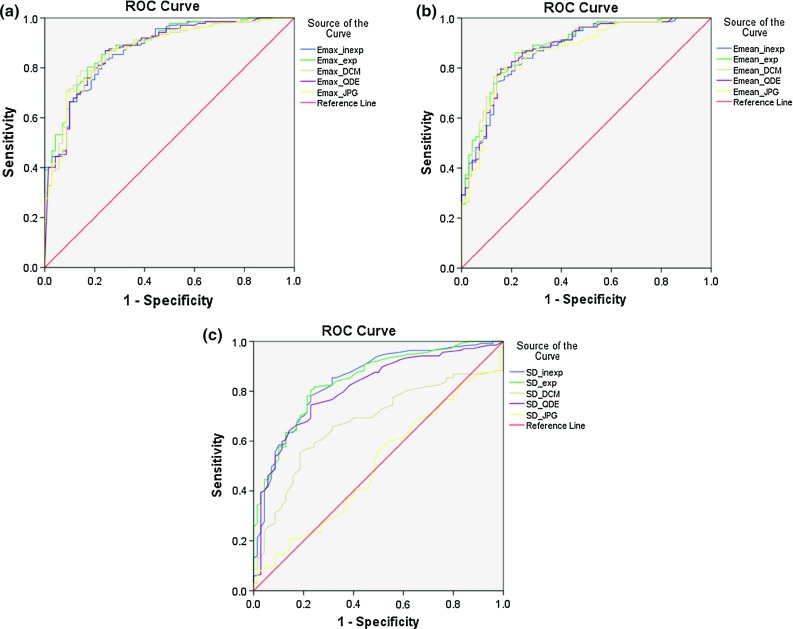
ROC analysis of ${E}_{\mathrm{max}}$ (**a**) and ${E}_{\mathrm{mean}}$ (**b**) gave similar performance for all formats while the performance of SD (**c**) was inferior for JPG and DICOM format than the manual estimation or QDE format

**Table 2:** Diagnostic performance and optimal cut-off thresholds estimated through Youden’s analysis for all formats

Analysis	AUC			Optimal cut-off thresholds [kPa]		
	${E}_{\mathrm{max}}$	${E}_{\mathrm{mean}}$	SD	${E}_{\mathrm{max}}$	${E}_{\mathrm{mean}}$	SD
Inexperienced observer	0.874	0.871	0.841	88	79	6.6
Experienced observer	0.888	0.871	0.844	91	70	8.1
CAD: DICOM	0.878	0.882	0.673	113	86	7.4
CAD: QDE	0.873	0.876	0.812	84	85	6.9
CAD: JPG	0.870	0.863	0.500	95	86	9.6

**Fig. 6:**
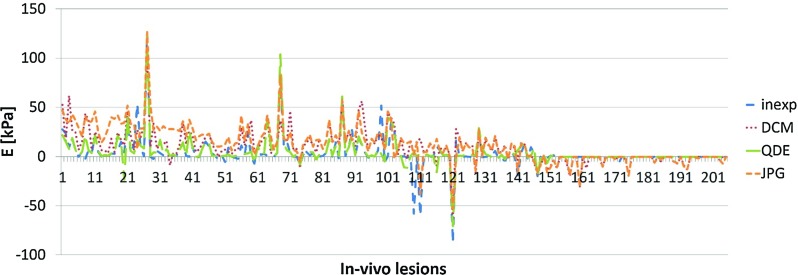
Difference in estimated values of ${E}_{\mathrm{max}}$ for the in vivo lesions; lesions sorted by stiffness, *each line* represents a lesion

**Fig. 7:**
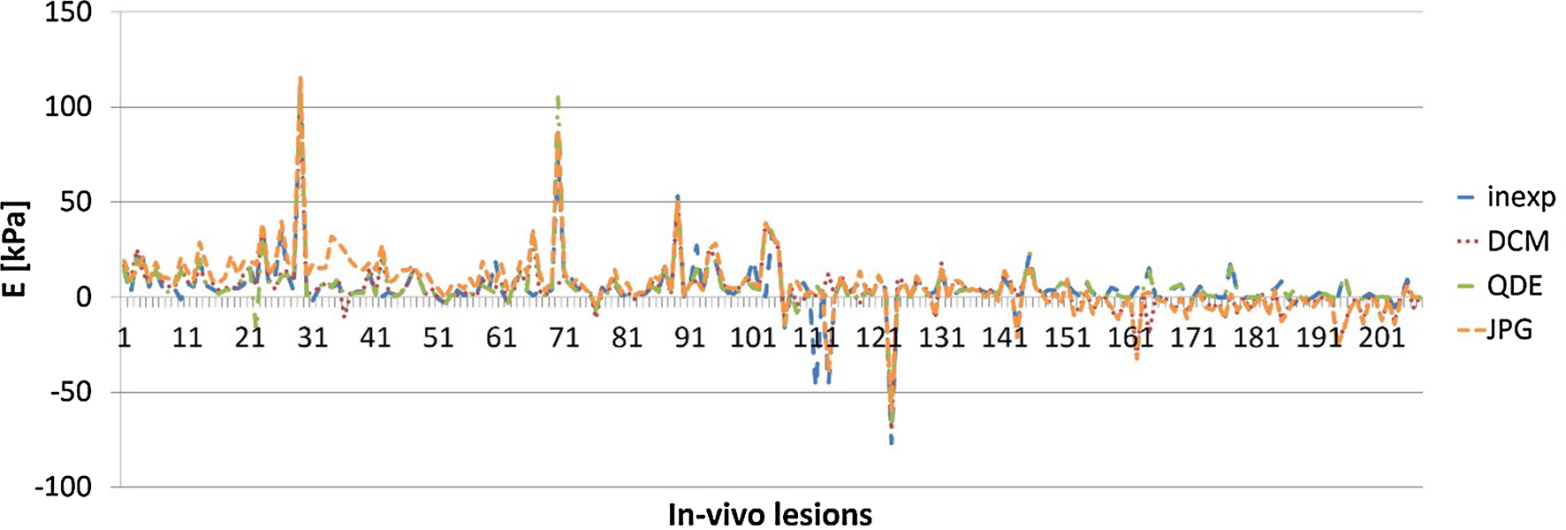
Difference in estimated values of ${E}_{\mathrm{mean}}$ for the in vivo lesions; lesions sorted by stiffness, *each line* represents a lesion

### Diagnostic performance in in vivo lesions

The diagnostic accuracy was not statistically significant different for all assessments (Table 1, *p* $\ge$ 0.1). However, the experienced observer achieved a significantly inferior sensitivity compared to the automatic assessment derived from images saved in DICOM ($p=0.049$) and JPG ($p=0.003$) when the threshold ${E}_{\mathrm{mean}}=50$ kPa was applied but a significantly superior sensitivity compared to the automatic assessment derived from images saved in DICOM when the threshold SD $=$ 7 kPa ($p=0.004$) was applied. The inexperienced observer achieved a significantly superior specificity compared to the automatic assessment derived from images saved in DICOM when the threshold SD $=$ 7 kPa was applied ($p=0.03$).

ROC analysis evaluating the influence of the quantification formats on the diagnostic performance of the elasticity parameters is shown in Fig. 5. The AUCs and the estimated optimal cut-off thresholds estimated by Youden’s Indices’ evaluation are shown in Table 2.

The diagnostic performance evaluated in terms of ${E}_{\mathrm{max}}$ (0.870 vs. 0.888) and ${E}_{\mathrm{mean}}$ (0.863 vs. 0.871) was similar for all formats. The performance of ${E}_{\mathrm{mean}}$ was for the DICOM format slightly superior to the manual observation (0.882 vs. 0.871). In terms of SD, the diagnostic performance of both the JPG and DICOM estimations was inferior to the other formats (0.673 vs. 0.844, 0.500 vs. 0.844). The estimated optimal cut-off thresholds for ${E}_{\mathrm{max}}$ and ${E}_{\mathrm{mean}}$ using DICOM and JPG images were higher than for the manual estimation, which agreed with the results of the numerical comparison. However, a difference can also be seen between the experienced and the inexperienced observer, which was in the same range as the difference between the automatic formats.

**Fig. 8:**
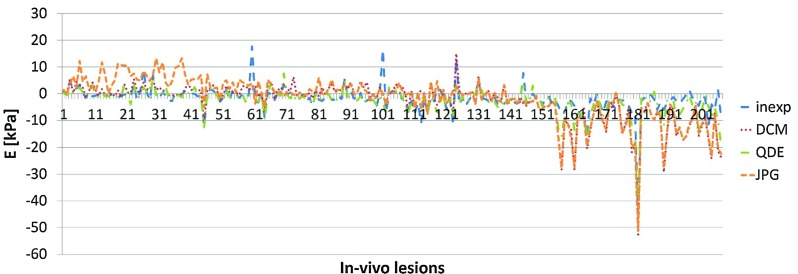
Difference in estimated values of SD for the in vivo lesions; lesions sorted by stiffness, *each line* represents a lesion

### SWE parameters in individual in vivo lesions

The experienced observer had the highest AUC. For a clearer representation of the results, the differences between each of the quantification formats and the experienced observer were evaluated. The lesions were first sorted regarding the maximum stiffness of their lesion, with one being the most compliant and 207 the stiffest. The differences between the averaged estimated values of ${E}_{\mathrm{max}}$ for all formats and the values for the experienced observer are shown in Fig. 6. The overall agreement of the different quantification formats was good. JPG estimations were higher for softer lesion (<45 kPa, lesion 41). The discrepancy between the formats was higher until patient 100, which equalled a stiffness of 105 kPa. The deviation was highest for the JPG format followed by the DICOM format. The values for the inexperienced observer and the QDE format were very similar. From patient 150, which equalled a stiffness of 165 kPa, i.e. close to the maximum stiffness visualised in the colour map, the difference between the formats was negligible. In individual lesions occurred larger discrepancies between the experienced and the other quantification formats.

**Table 3:** ICC analysis for all formats gives very good agreement for all quantification modes for ${E}_{\mathrm{max}}$ and ${E}_{\mathrm{mean}}$ and a poor agreement for SD

	Experienced			DICOM			QDE			JPG		
	${E}_{\mathrm{max}}$	${E}_{\mathrm{mean}}$	SD	${E}_{\mathrm{max}}$	${E}_{\mathrm{mean}}$	SD	${E}_{\mathrm{max}}$	${E}_{\mathrm{mean}}$	SD	${E}_{\mathrm{max}}$	${E}_{\mathrm{mean}}$	SD
inexp	0.977	0.982	0.854	0.978	0.990	0.692	0.992	0.995	0.918	0.972	0.991	0.494
exp	–			0.966	0.984	0.501	0.979	0.981	0.808	0.950	0.973	0.293
DCM				–			0.985	0.992	0.826	0.985	0.991	0.879
QDE							–			0.976	0.992	0.636

Figure 7 shows the difference between the experienced observer and the other formats for ${E}_{\mathrm{mean}}$. The agreement was generally superior to that for ${E}_{\mathrm{max}}$. The overall agreement of the different formats was good. However, larger discrepancies occurred in individual lesions. The difference between the different formats was negligible, and most peaks were in correspondence for all formats as they were for ${E}_{\mathrm{max}}$. The peaks also occurred for the same lesions.

The differences between the estimated values of SD for the experienced observer and all other formats are shown in Fig.8. The overall agreement was good for a moderate stiffness (48–175 kPa, lesions 45–151). SD was larger for the JPG estimation than the other formats in softer lesions; the estimations from JPG and DICOM format were smaller than the other formats in harder lesions. Small deviations of the values with the JPG format occur until lesion 45, equal to ${E}_{\mathrm{max}}=48~\hbox {kPa}$, and from lesion 151, equal to ${E}_{\mathrm{max}}=175~\hbox {kPa}$. The discrepancy for the JPG format for stiffer lesions is similar to that for the DICOM format. This is caused by the truncated range of the colour map ending at 180 kPa in contrast to the data in the imaging system (manual observation and the QDE format) which ranges up to 300 kPa, as previously noted. Hence, minor differences in the elasticity in this range occur in the saved images. An adjustment as for ${E}_{\mathrm{max}}$ and ${E}_{\mathrm{mean}}$ was not applied.

### ICC analysis

The ICC comparing all formats was 0.990 for ${E}_{\mathrm{max}}$, 0.982 for ${E}_{\mathrm{mean}}$ and 0.861 for SD. Table 3 shows the agreements for the different formats with each other. The agreement between the inexperienced observer and the QDE estimation was nearly perfect (0.992, 0.995, 0.918). The SD values agreed poorly for the DICOM and JPG formats.

## Discussion

In this paper, we have introduced an easy and reproducible algorithm to automatically estimate the elasticity parameters from SWE images saved in DICOM, JPG and QDE format (for research only and not available on the commercial devices), to enable remote quantitative evaluation of SWE images obtained from the Aixplorer ultrasound imaging system according to the applied clinical evaluation. While an adjusted colour map had to be used, the results of the automatic estimation were in agreement with manual estimation. Thus, the proposed algorithm is suitable to enable long-term studies of images saved on the PACS system offline of the ultrasound device. As the introduced algorithm evaluates SWE images accordingly to the clinical procedure, the algorithm also supports multi-centre studies eliminating an inconsistent imaging protocol.

We evaluated images saved in the formats DICOM and JPG as these are the default saving formats at the device. One could argue that the saving format theoretically should not affect the diagnostic performance. However, the JPG format uses lossy compression by default using the pre-settings at the device. Although this is possible to adjust before saving, we did not change the pre-settings as our aim was to include images saved more than 5 years earlier, i.e. qualifying to be included in long-term studies. These images are most likely saved using the default pre-settings. Our study aims to proof the clinical validity to use both saving formats for the automatic image assessment. We included the QDE format as gold-standard for the quantitative conversion. In addition, including the QDE format allows direct comparison of the automatic and the manual assessment to proof validity of the general performance of the automatic evaluation.

Very good reproducibility was achieved for ${E}_{\mathrm{max}}$ and ${E}_{\mathrm{mean}}$. However, the reproducibility of the SD values was not satisfactory, which we hypothesise is because of smoothing of the SWE image applied by the ultrasound device prior display. Furthermore, we suggest that image compression has an influence on the agreement, as the performance with JPG images, which undergo lossy compression in the default setting, was inferior to that with DICOM and even more so with QDE or manual assessment. Overall the agreement of all quantification formats compared with the experienced observer is better for ${E}_{\mathrm{mean}}$ than for ${E}_{\mathrm{max}}$.

No statistical significant difference in the diagnostic performance was observed if the threshold ${E}_{\mathrm{max}}=80~\hbox {kPa}$ was applied. The automatic assessment derived from images saved in DICOM and JPG format gave a superior sensitivity compared to the assessment by the experienced observer. This is probably caused by the slightly higher elasticity values acquired from DICOM and JPG images as observed in the phantom study. The diagnostic performance of the automatic assessment derived from DICOM images was inferior to the manual assessment when SD was evaluated, which is probably caused by the smoothing applied as discussed above.

Although the agreement of the different image evaluations was generally very good, occasional large discrepancies occurred. These are likely to be caused by differences in the exclusion of artefacts, which is still done manually, in this study by the inexperienced observer. This also leads to better agreement of the performance between the inexperienced observer and the QDE values (0.992, 0.995, 0.918) than between the manual estimations by the inexperienced and experienced observers (0.977, 0.982, 0.854). This indicates that the algorithm could be improved by developing a method of automatic exclusion of artefacts.

There are several imaging modalities, such as computer tomography (CT) and magnet resonance imaging (MRI), where quantitative parameters are extracted from images within standard care procedures to allow post-processing analysis [9–13]. Correspondingly, the algorithm introduced in this paper enables post-processing analysis of SWE images offline from the imaging system itself, allowing this operation to be performed wherever and whenever the observer chooses, e.g. in their office. Extraction of quantitative data from PACS is possible, allowing a direct comparison of data from multiple imaging modalities, and this will be especially important if SWE is going to be accepted as standard care.

The introduced algorithm might also support to define a standard for the clinical application of SWE. The used ROI size is easily adjustable by the observer, and also the evaluation of the various elasticity parameters is possible without human bias. Furthermore, the algorithm also allows the analysis of images taken at different times and at different sites. This would enhance the validity of multi-centre studies with images analysed with identical procedures. Although SWE has been shown to be highly reproducible [2, 29], there is still no standard defined for its performance and evaluation and differences in technique and evaluation will continue to make comparison between studies difficult. A further benefit of the algorithm is that it avoids observer bias from the appearance of the lesion on the greyscale image, making SWE analysis more objective.

In future, the algorithm for the automatic evaluation of elasticity parameters can be further improved. First, an automatic exclusion of artefacts is necessary to allow full automation of the process and manual validation by an observer will not be needed. Furthermore, this will also reduce the run-time of the overall procedure. Hence, the algorithm should be trained to exclude the pectoral muscle, skin and artefacts, e.g. reflections of stiffness radiating from the skin (stripe pattern).

The discrepancy between the inexperienced and the experienced observers, which is also reflected in the automatically calculated results, suggests that the experience of the observer may be important but could also represent bias of the experienced observer from the greyscale appearances of the lesions. The ability of the algorithm to achieve a performance as good as that of an experienced observer is promising. This will save time and therefore reduce the cost of future assessment of breast lesions.

Finally, automatic analysis of qualitative characteristics such as the Tozaki pattern [30] might be of interest as introduced by Skerl et al. [24]. Combination of this algorithm and an automatic BI-RADS assessment would enable fully automated assessment of ultrasound images of solid breast lesions.

This study has limitations as it was a single-centre, retrospective study. The observers were blinded to the final pathology of the lesions to minimise bias, but they were aware of the patients’ ages and the greyscale appearance of the lesions.

In conclusion, this paper has reported a first step in the development of SWE CAD. Taking the adjusted colour map into account, the analysis of SWE images saved with an opacity of 50% in DICOM or JPG is possible. This allows quantitative image analysis of all images saved at any time from the device such as images stored on the hospital’s PACS. Therewith the introduced algorithm enables image evaluation of all saved images comparable to the assessment at the device. Long-term studies of images obtained more than five years previously are feasible allowing a flexible image evaluation to study novel characteristics and to apply novel procedures. Likewise, multi-centre studies are feasible with the introduced algorithm even if an inhomogeneous image evaluation protocol was applied at the participating sites. However, images saved in DICOM achieve a superior agreement with the manual estimation and should therefore be preferred.
